# Scoping review on natural language processing applications in counselling and psychotherapy

**DOI:** 10.1111/bjop.12721

**Published:** 2024-08-02

**Authors:** Maria Laricheva, Yan Liu, Edward Shi, Amery Wu

**Affiliations:** ^1^ Educational and Counselling Psychology, and Special Education The University of British Columbia Vancouver British Columbia Canada; ^2^ Psychology Carleton University Ottawa Ontario Canada; ^3^ Arts, Business and Law Victoria University Melbourne Melbourne Victoria Australia

**Keywords:** bias, counselling, generalizability, natural language processing, psychotherapy, scoping review

## Abstract

Recent years have witnessed some rapid and tremendous progress in natural language processing (NLP) techniques that are used to analyse text data. This study endeavours to offer an up‐to‐date review of NLP applications by examining their use in counselling and psychotherapy from 1990 to 2021. The purpose of this scoping review is to identify trends, advancements, challenges and limitations of these applications. Among the 41 papers included in this review, 4 primary study purposes were identified: (1) developing automated coding; (2) predicting outcomes; (3) monitoring counselling sessions; and (4) investigating language patterns. Our findings showed a growing trend in the number of papers utilizing advanced machine learning methods, particularly neural networks. Unfortunately, only a third of the articles addressed the issues of bias and generalizability. Our findings provided a timely systematic update, shedding light on concerns related to bias, generalizability and validity in the context of NLP applications in counselling and psychotherapy.

## BACKGROUND

Natural language processing (NLP) is an interdisciplinary field that combines linguistics with computer science within the realm of artificial intelligence (Joshi, 1991). Artificial intelligence (AI) refers to the simulation of human intelligence processes by a system or a machine (Russell & Norvig, 2016; Xu, Liu, et al., 2021). AI includes a broad range of applications with different focuses, including NLP, machine learning, computer vision, robotics and decision‐making systems. NLP specializes in text analysis. Over the past decade, NLP studies have incorporated many machine learning methods, such as regression, neural networks and clustering, due to the advancement of techniques for effectively quantifying text data.

More specifically, the goal of NLP is to get computers to perform practical tasks involving human language such as speech recognition, information extraction and language generation (Jurafsky & Martin, 2000). One of the earliest applications of NLP was in the context of counselling. In 1966, Joseph Weizenbaum created Eliza, a chatbot simulating a psychotherapist who engaged in conversations with humans (Weizenbaum, 1966). The evolution from Eliza to contemporary NLP applications underscores the fundamental impact of artificial intelligence in the realms of health and well‐being. Given the rapid growth of this subject area, it is crucial for psychologists and researchers in related fields to have a better understanding of the applications of NLP in counselling and psychotherapy. This study aims to provide a scoping review of these NLP applications.

Recent years have witnessed a significant increase in the number of NLP studies in health and well‐being research. For example, NLP has been used to improve patients' psychological well‐being by assisting in the diagnostics of mental disorders and personalizing pharmacological treatment (Su et al., 2020). Electronic health records (EHR), such as clinical notes, reports and treatment plans, are the main source of NLP data for mental health research (Shatte et al., 2019; Spasic & Nenadic, 2020). There are many advantages of EHRs: being structured, low cost and in large amounts. These advantages helped identify meaningful patterns in texts and enhanced performance in prediction tasks, for example, diagnoses (Pavlova & Uher, 2020; Smoller, 2018).

NLP methods are useful for psychotherapy research due to their ability to analyse and extract key information from text data, such as session transcripts, treatment notes and patient reports. Unlike conventional methods, that is, qualitative data analytical methods, NLP allows researchers to process and analyse vast amounts of text data efficiently and handle complex linguistic structures, including syntax, semantics and context. Below, we reviewed some major areas where NLP is applied in psychotherapy research.

In counselling and psychotherapy, NLP technologies have been implemented in four main areas: outcome prediction, monitoring the counselling process, automated coding of counselling conversational data and investigation of language patterns. *Outcome prediction*, including diagnosis and prognosis, is the most common application of machine learning in mental health research (Shatte et al., 2019). However, the outcome prediction is often compromised by the fact that data are mostly counselling transcripts that are messy (unstructured) and difficult to quantify. Therefore, instead of aiming for specific diagnoses, NLP in counselling is often used to identify ‘signs’ of mental health problems (Fitzpatrick et al., 2017; Goldberg et al., 2020). For example, NLP has been used to examine the association of language patterns with conversational outcomes (Burkhardt et al., 2021) or treatment outcomes assessed by psychological scales (Althoff et al., 2016). By using NLP methods, Althoff et al. (2016) identified effective conversational tactics that counsellors implemented, such as adaptability and creativity, and demonstrated that the use of NLP allowed them to analyse large‐scale data (more than 3 million text messages).

*Monitoring the counselling process* is another common application of NLP. When data are collected over time, NLP models can track a patient's progress in therapy. NLP algorithms can detect linguistic changes and identify the association between these changes with fluctuations in symptoms. This approach to monitoring progress is not subject to prior beliefs or theories but relies on objective analysis of the language (Shatte et al., 2019). Previous works showed that NLP could successfully identify common trends and patterns based on the conversations in counselling sessions (Goldberg et al., 2020). One of the implementations of NLP for monitoring purposes is illustrated by Tay's (2020) article in which Tay demonstrated how therapy sessions can be classified into four sequential clusters by analysing the language style. Moreover, Tay also showed that this automated approach was compatible with the traditional approach and claimed, ‘having determined the clusters, we may choose to qualitatively scrutinize them in different ways’ (p. 21).

*Automated coding* is widely used in counselling and psychotherapy applications. Researchers often label conversational data based on a pre‐defined coding system. In NLP, each conversational turn is called an utterance. Some studies have shown that coding at the utterance level can be automated by NLP, even though the accuracy is not yet comparable to that of human annotators (Can et al., 2016; Laricheva et al., 2022; Tanana et al., 2016). Nevertheless, automated coding of utterances using NLP can help identify clients' cognitive and emotional states much faster than humans and provide a general overview for researchers (Bantilan et al., 2020; Can et al., 2016; Gibson et al., 2017; McCarthy et al., 2017; Syzdek, 2020; Tanana et al., 2016). In other situations, researchers may prefer to use a single code for an entire counselling session rather than codes for each utterance. A session code can be an indication of a therapist's level of adherence, or a representation of the main topic/theme discussed in a counselling session (Atkins et al., 2012; Can et al., 2016; Flemotomos et al., 2021). For example, Atkins et al. (2012) demonstrated how topic models could summarize key themes in a couple's course of therapy and highlighted that, unlike other methods, topic models were not restricted by pre‐determined categories (p. 13).

NLP methods also allow for quantitative *investigation of language patterns*, enabling researchers to identify and measure linguistic features associated with therapeutic processes, treatment outcomes and patient characteristics. Such analysis can lead to a better understanding of the linguistic aspects of psychotherapeutic interactions. For example, Qiu and Tay (2021) examined changes in language use across therapeutic roles and demonstrated that NLP methods efficiently analysed large‐scale data, allowing for an expanded research scope beyond what traditional discourse analysis permits (p. 1).

Efficiency and objectivity are the most mentioned advantages of NLP use for psychotherapy research (Althoff et al., 2016; Can et al., 2016; Tanana et al., 2016). NLP can also be useful for exploratory analysis that uncovers trends and patterns in the data (Imel et al., 2015). However, the researchers face several challenges when applying NLP. First, counselling transcripts tend to be context dependent and lack a clear structure for NLP analysis, which often affects prediction accuracy (Calvo et al., 2017). Additionally, counselling data are expensive due to the labour‐intensive transcription process (Tanana et al., 2016). As a result, most previous studies were based on small samples (Althoff et al., 2016).

Additionally, several ethical issues have been raised regarding the use of NLP in counselling and psychotherapy (Goirand et al., 2021; Joerin et al., 2020; Kretzschmar et al., 2019; Luxton et al., 2016). The major concerns reside in the generalizability of results and the risk of biases (Aafjes‐van Doorn et al., 2021; Joerin et al., 2020). Although not directly involving human subjects, NLP can negatively affect individuals' lives if decisions (e.g. allocation of resources) are made based on the outcomes of a poor application. The situation can become worse if a flawed NLP application contributes to systematic unfairness, particularly affecting a subpopulation or specific group (Blodgett et al., 2020). In health care, for instance, some applications have been shown to introduce bias against gender (Agmon et al., 2022) and disability groups (Hutchinson et al., 2020). In mental health, Straw and Callison‐Burch (2020) showed that NLP could introduce or aggravate biases related to age, religion, race and nationality.

To understand bias, in this review, we followed the framework described by Hovy and Prabhumoye (2021). It defines five types of bias occurring at consecutive steps of model development. One source of bias is the choice of data or selection bias (Shah et al., 2020). Selection bias arises when the samples chosen for the model training are non‐representative of the target population for which the model is intended. Selection bias causes poorer model performance for non‐represented demographic groups, such as African American speakers (Hovy, 2015). Bias can also be introduced through annotations, which is referred to as label or annotation bias. This type of bias arises when annotators systematically choose the wrong labels due to the lack of training or opposing views. The third type of bias, originating from input representations (e.g. word embeddings and the representations of words in numeric space) is called semantic bias. Some word embedding systems were shown to reflect historical biases such as gender and racial biases (Basta et al., 2019; Bhardwaj et al., 2021; Straw & Callison‐Burch, 2020; Zhao et al., 2019). The fourth type of bias is research design bias which occurs when the system is applied in a context different from the one it was originally designed for. Additionally, NLP models themselves may compound the bias that already existed in the system, called bias overamplification. The reason for that bias compound is that the algorithm detects correlations from biased training data and uses them to improve precision (Hovy & Prabhumoye, 2021, p. 9).

Several reviews have discussed NLP applications in mental health. However, none of them provided a comprehensive, systematic review, specifically for counselling and psychotherapy. For example, Straw and Callison‐Burch (2020), Glaz et al. (2021) and Su et al. (2020) reviewed papers that applied machine learning and NLP to mental health. However, none of the reviews included studies based on client therapy conversations; instead, these reviews focused on medical records (Spasic & Nenadic, 2020). Only one review examined the use of non‐clinical text on social media (Calvo et al., 2017).

Our study aims to fill this gap, providing general guidance for researchers to apply NLP methods more appropriately and facilitating the development of more practical NLP methods for analysing counselling conversational data. The purpose of this study is twofold. The first purpose is to review how NLP methods have been applied in counselling and psychotherapy from 1990 to 2021 and to identify the challenges, advancements, limitations and research gaps in these studies. The second purpose is to investigate how the issues of bias and generalizability have been addressed in NLP applications for counselling and psychotherapy. Specifically, we addressed the following research questions:

RQ1: What were the general trends of NLP applications (i.e. publication count, study purpose and type of data used) in counselling and psychotherapy research from 1990 to 2021?
RQ2: How did researchers implement NLP in these applications? More specifically, we looked into the specific NLP methods (models and algorithms), model validation approaches and the measures used to evaluate NLP performance.
RQ3: How did researchers evaluate the generalizability of their NLP results?
RQ4: How did researchers evaluate the potential biases in NLP applications?

## METHODS

To address these research questions, we conducted a scoping review (Aafjes‐van Doorn et al., 2021; Shatte et al., 2019) using peer‐reviewed journal articles that applied NLP to counselling and psychotherapy and were published between 1990 and 2021. We chose to include papers starting from 1990 because the methodology of NLP was rapidly developed and became an independent topic area in computer science in the early 1990s. This scoping review followed the guidelines of preferred reporting items for systematic reviews and meta‐analysis (PRISMA) provided by Page et al. (2021).

### Search strategy

We conducted a keyword search in three academic databases, including Web of Science, PsycINFO and PubMed. We searched the articles with the following keywords (and their variations) in the titles and abstracts: (a) psychotherapy (‘psychotherapy*’, ‘counselling’, ‘counsellor*’, ‘counselling’, ‘counsellor*’, ‘mental health’ and ‘mental disorders’); (b) transcripts (‘transcript*’, ‘recording*’, ‘conversation*’, ‘utterance*’, ‘session*’ and ‘interaction*’); and (c) NLP (‘NLP’, ‘natural language processing’, ‘machine learning’, ‘ML’, ‘artificial intelligence’, ‘AI’ and ‘neural network’). The publication date was restricted to the period of 1990 to 2021. The data were extracted on 25 September 2022.

### Inclusion and exclusion criteria

Only peer‐reviewed journal articles in English were included in this study. The included studies had to meet the following criteria: (1) utilized psychotherapy‐related textual data (e.g. transcripts of traditional counselling, online chats from text‐based counselling or recordings of hotline counselling calls); (2) included interactive conversations between a support seeker (e.g. a client/patient) and a support provider (e.g. therapist/counsellors/peer supporters); and (3) applied at least one NLP method. We excluded qualitative studies, systematic reviews and pure methodology and feasibility^1^ studies. We also excluded articles that used narrative data (e.g. psychotherapy notes or thought records), social media data, EHRs or conversations outside the counselling context. Lastly, we excluded studies that did not clearly specify their NLP methods.

**FIGURE 1:**
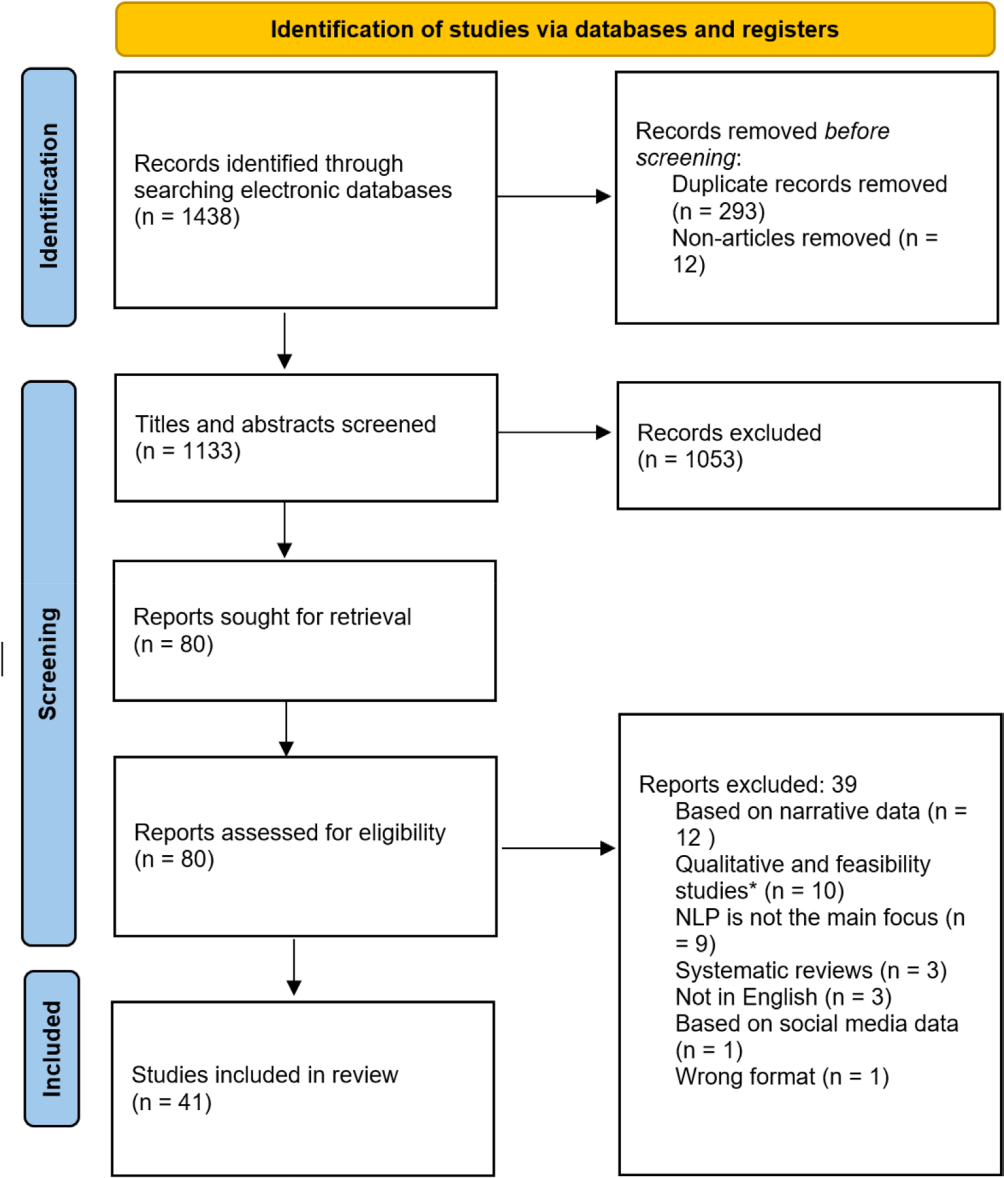
Flowchart of systematic search procedures. *Note*: The flowchart was adapted from the template provided by Page et al. (2021).

Our search strategy resulted in 1438 papers. After removing duplicates and non‐journal articles, a total of 1133 papers were included for further screening. The titles and abstracts of the article were first screened by two independent reviewers (graduate students trained in NLP and systematic review) using Rayyan, an online software for performing systematic reviews (Ouzzani et al., 2016). Figure 1 depicts the whole process of our article search and selection.

Of 1133 papers, the two reviewers had disagreements on 64 (5.6%) articles. The disagreements were resolved in consultation with a senior researcher. After resolving the disagreements, 80 articles were retained for further full‐text screening. After the full‐text screening, 39 papers were removed due to using the wrong format of data (narrative instead of conversational), being a qualitative study, applying NLP as a secondary method or being written in a different language or a different format. A total of 41 articles were kept for this review.

### Data analysis

The extracted data were analysed in *R* using the *dplyr* package (Wickham et al., 2023) for data manipulation and the *ggplot2* package (Wickham, 2016) to create graphs and charts.

### Transparency and openness

We provide a full description of the data used in this study. Since we used the data publicly available online, REB approval is not requested.

## RESULTS

The Results section is organized by four research questions, including the general trends of NLP applications, the implementation of NLP methods and the evaluation of generalizability and bias issues. The detailed results are summarized using a table format and can be found in the Appendix A.

### RQ1: General trends of publications

No eligible articles were found between the years 1990 and 2004. Since 2005, the number of peer‐reviewed journal articles has been increasing, especially in the last few years of the study period. The size of the transcripts included in the studies varied greatly, ranging from fewer than 10 sessions to more than 1000 sessions. We also identified four common purposes for applying NLP: automated coding, outcome prediction, counselling session monitoring and language pattern detection. In what follows, we provided more details about the trends, including the increase in the publication count, study purposes and type of data for applying NLP.

#### Publication count over time

**FIGURE 2:**
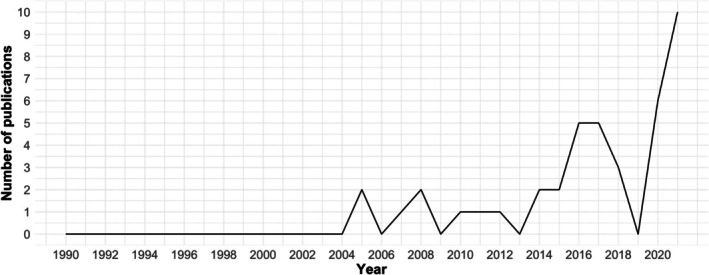
Number of publications that applied NLP over time (1990–2021).

Figure 2 shows the publication count over the studied period. The articles that met our screening criteria first appeared in 2005. The number of papers increased rapidly in the last 2 years with 16 (39%) published between 2020 and 2021. The notable surge in publications could be attributed to the increased availability and accessibility of NLP tools.

#### Study purposes for applying NLP

Of 41 articles, 20 articles (49%) aimed at automating the coding process; 12 articles (29%) intended to predict patients' treatment outcomes or therapeutic changes; 7 (17%) focused on monitoring the counselling process through language changes; and 5 (12%) investigated language patterns in counselling sessions. A detailed description of study purposes for each article can be found in the Appendix A.

##### Automated coding

Among 20 automated coding articles, 14 (70%) conducted utterance‐level analysis and 6 (30%) conducted session‐level analysis. For the utterance‐level analyses, the coding could be for a single or multiple classes. Six of these fourteen utterance‐level studies performed a single‐class coding. The single‐class classification was used to identify the occurrence of a particular event in an utterance, such as affect expression (Halfon et al., 2021), humour (Ramakrishna et al., 2018), traumatic memory reactivation (Wiegersma et al., 2020), suicide thoughts (Bantilan et al., 2020; Xu, Xu, et al., 2021) and a mention of a significant event (McCarthy et al., 2017). Eight of fourteen articles on utterance coding conducted a multiclass classification. Multiclass coding has been conducted to distinguish among therapy stages (Nitti et al., 2010), therapy approaches (Imel et al., 2015), emotions (Tanana et al., 2021) and sentiment expressions (Syzdek, 2020). Instead of developing coding schemes for their data, four articles adopted previously established coding schemes; three articles (Can et al., 2016; Tanana et al., 2016; Tavabi et al., 2020) used the verified versions of the Motivational Interviewing Skills Code (Miller et al., 2003); and one article (Goldberg et al., 2020) used the Working Alliance Inventory (Hatcher & Gillaspy, 2006).

As for the six articles that focused on session‐level automated coding, one article (Flemotomos et al., 2021) predicted the total score of the Cognitive Therapy Rating Scale (CTRS; Young & Beck, 1980) and categorized the scores as low or high CTRS; another article (Xiao et al., 2016) evaluated therapists' skills by assigning a code to each session. The remaining four articles used topic modelling techniques to summarize themes discussed in a therapy session (Atkins et al., 2012, 2014; Atzil‐Slonim et al., 2021; Gaut et al., 2017).

##### Outcome prediction

Twelve articles investigated the relationship between linguistic features and therapeutic outcomes. A variety of strategies were used to generate the outcome variables for NLP analysis. Ten of twelve used patients' self‐report assessments. In two articles, raters were used to quantify the outcome. Kahn et al. (2008) used external raters to evaluate the smoothness and depth of the session, and Fontao and Mergenthaler (2008) rated the outcome themselves. Some researchers used other methods to measure therapeutic outcomes. Among them, two articles used treatment adherence (specifically, abstinence from alcohol) as the outcome measure (Rentscher et al., 2017; Soriano et al., 2017). One article (Sonnenschein et al., 2018) used structured clinical interviews (Structured Clinical Interview for Axis I DSM‐IV Disorders; First et al., 1995) to measure the therapeutic outcome.

##### Monitoring a counselling session

Seven articles focused on monitoring the dynamic processes in psychotherapy. These articles attempted to describe how specific linguistic indicators (e.g. words to express affective processes) changed throughout a therapy session. However, this task was a secondary objective in two studies. Syzdek (2020) first categorized the sentiment for each utterance and then evaluated its change over time. Burkhardt et al. (2021) focused on outcome prediction and examined the correlation between language indicators and scores on the Patient Health Questionnaire.

##### Investigating language patterns

Five articles pursued the broader goal of investigating language patterns. Two articles compared how language use changed depending on therapeutic roles (Qiu & Tay, 2021) or emotional–cognitive regulation (Tonti & Gelo, 2016). The other three articles focused on different aspects of language patterns. Lepper and Mergenthaler (2005) explored the language features corresponding to the occurrence of cohesion phenomena in group psychotherapy. Cariola (2015) compared the following two dictionaries: the use of words related to barrier imagery and the use of words associated with psychological processes. The researchers attempted to understand the correlation between the uses of these dictionaries. Hull et al. (2021) identified the major themes and symptom clusters in text‐based counselling.

#### Type of data used

Among 41 articles, 35 (85%) utilized counselling session transcripts, and 6 (15%) used conversation histories derived from text‐based counselling services. Among the articles that used counselling session transcripts, 10 utilized large data sets that could be provided by a subscription or a special request, for example, Beck Community Initiative (Flemotomos et al., 2021) or Alexander Street Press data (Gaut et al., 2017; Imel et al., 2015; Tanana et al., 2021). Fifteen articles used private data that were collected by other organizations or researchers; seven researchers collected data themselves, with the sizes of the data sets ranging from 5 sessions (Cardazzone et al., 2021) to 1486 sessions (Atkins et al., 2012). Due to privacy concerns, none of the authors have published their data.

Most of the researchers utilized individual counselling sessions. However, Lepper and Mergenthaler (2005) explored the processes that occurred during group therapies, and Rentscher et al. (2017) and Soriano et al. (2017) used data from couple therapy. Instead of traditional counselling sessions, Halfon et al. (2021) collected transcripts from play therapy^2^ sessions since their participants were children.

A relatively new format of counselling that gained more popularity during the COVID‐19 pandemic is text counselling. Six articles (15%) used data collected from online telemedicine therapy platforms, such as Talkspace (Bantilan et al., 2020; Hull et al., 2021) or OpenUp (Xu, Xu, et al., 2021). Given the flexibility of the online format, interactions on these platforms differed from those in conventional therapy as one counsellor may chat with multiple patients simultaneously due to high demand during the pandemic. Additionally, certified counsellors on such platforms might be substituted by trained volunteers (Xu, Xu, et al., 2021).

### RQ2: Implementation of NLP methods and validation strategies

The review of the methods was done separately for basic automated text analysis and more advanced methods that require the knowledge of other major software (e.g. Python and R).

#### Automated text analysis tools

Automated text analysis tools directly process textual data and do not require any prior data transformations. These programs analyse text and compute word counts for each semantic category (e.g. words related to affective processes or anxiety). Eighteen articles (44%) used basic automated text analysis, such as Linguistic Inquiry and Word Count (LIWC) to obtain word counts. Of these 18, 8 articles used LIWC, 7 used the therapeutic cycle model (TCM; Mergenthaler, 1996) and 3 used both. Seven articles used other tools, including computerized reflective functioning (CRF; Boldrini et al., 2018) and W‐matrix, which is an improved version of LIWC (Soriano et al., 2017). The methods(s) used for each article were provided in the Appendix A.

**FIGURE 3:**
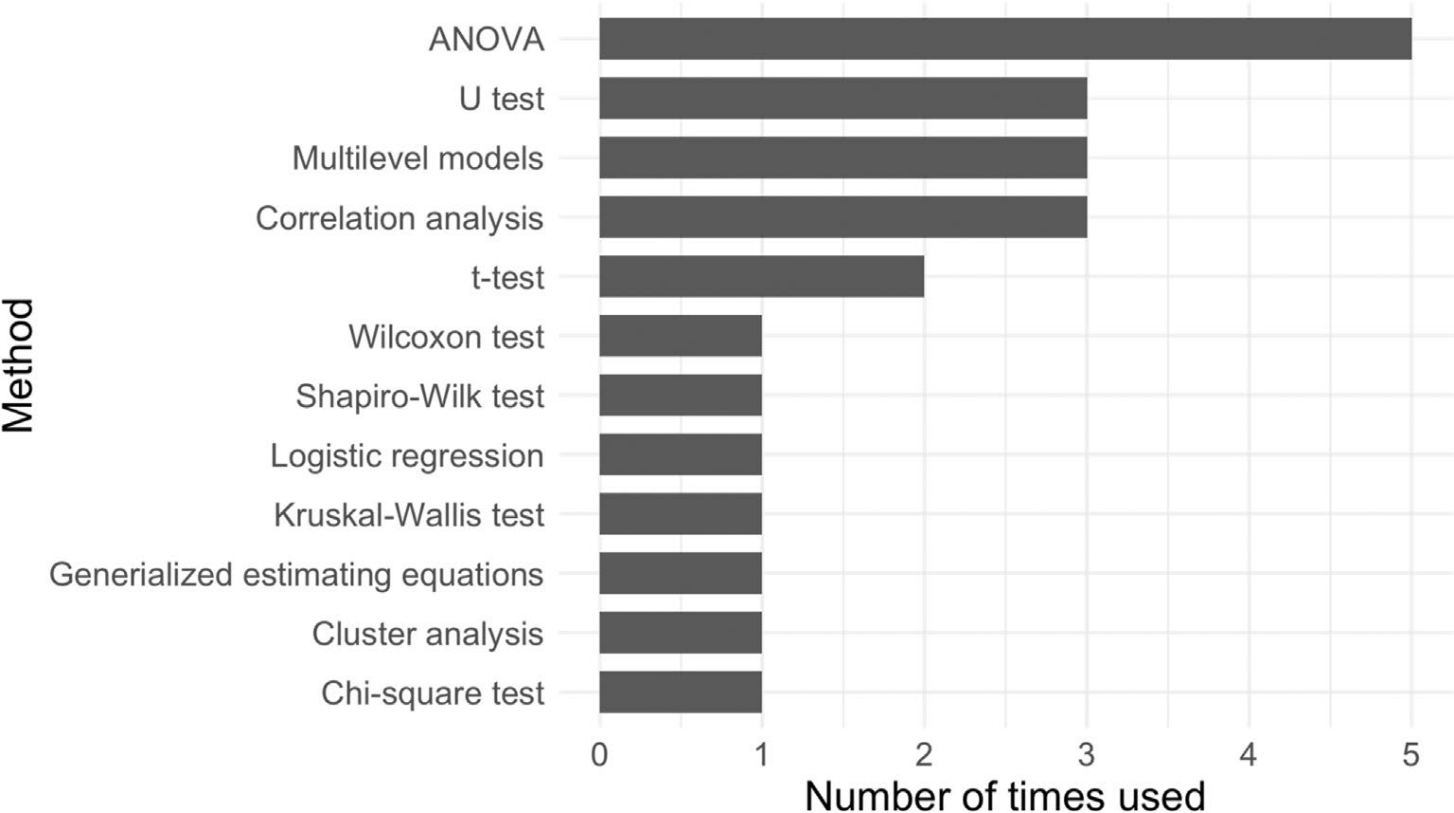
Statistical methods used in 18 articles that adopted automated text analysis tools.

Quantitative data obtained from automated text analysis tools can serve as input for further statistical analysis and hypothesis testing. Figure 3 lists all the statistical methods used in these kinds of studies. Eighteen articles reported statistical significance with *p*‐values. Among them, five articles conducted ANOVAs, three used multilevel models and three used correlation analysis. Other articles used statistical methods, such as *t*‐test, Wilcoxon test, Shapiro–Wilk test, Kruskal–Wallis test, logistic regression, generalized estimating equations, cluster analysis and Chi‐square test. Six of the eighteen articles provided corresponding effect sizes, predominantly Cohen's *d*. Three articles used *R*‐squared as an evaluation metric.

#### Advanced methods and techniques

Twenty‐three (56%) of the articles included in this review employed more advanced techniques. Among these articles, 16 applied supervised learning techniques, with neural networks being the most reported method. Twelve articles implemented cross‐validation techniques to test the model's ability to predict new data. Fourteen articles reported standard measures, such as F1 or accuracy, for evaluating classification performance. The rest of this section gave more details about these more advanced methods.

##### Word embeddings

Because most machine learning methods can only take numerical data as input, raw text data need to be transformed into numbers by techniques like word embedding before it can be used in machine learning (Joshi, 1991). Among the 23 articles, 6 applied the term frequency‐inverse document frequency (TF‐IDF; Chowdhury, 2005) technique. Three articles used the bidirectional encoder representations from transformers (BERT; Devlin et al., 2019). Four articles used other techniques, such as Global Vectors for Word Representation (GloVe; Pennington et al., 2014), word2vec (Mikolov et al., 2013) and sent2vec (Pagliardini et al., 2018). One article created its own custom word embeddings utilizing the long short‐term memory networks model (Ramakrishna et al., 2018).

##### Supervised and unsupervised learning

**FIGURE 4:**
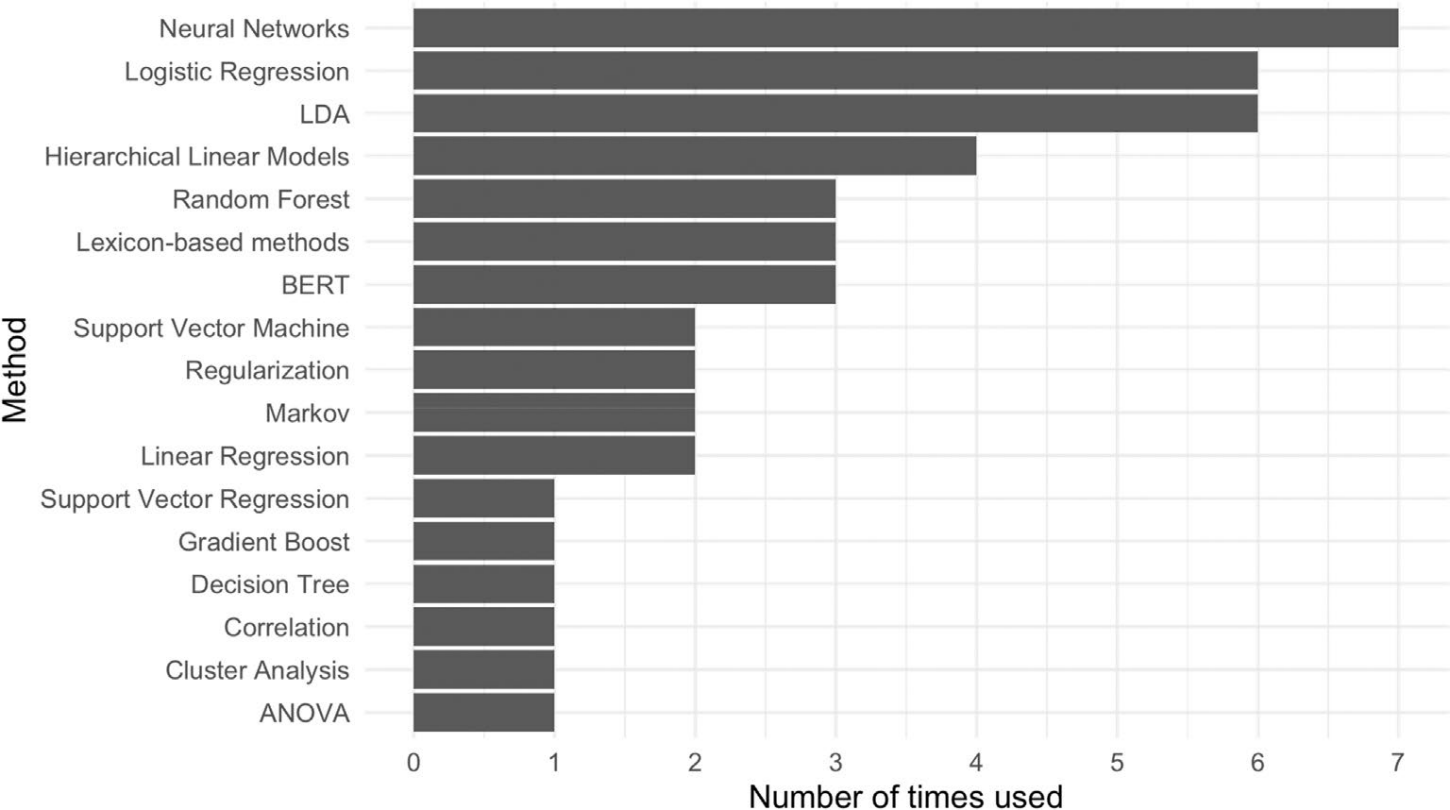
Statistical methods used with advanced machine learning techniques. *Note*: LDA stands for latent Dirichlet allocation; BERT for bidirectional encoder representations from transformers (BERT); and ANOVA for analysis of variance. It should be noted that hierarchical linear models and ANOVA are inferential statistics, which are conducted with the input data obtained from machine learning methods.

Twenty‐three (56%) of the articles applied supervised and unsupervised machine learning to perform classification and prediction tasks. Figure 4 lists the various machine learning methods and the frequencies being used. In what follows, we provided a brief description of supervised and unsupervised machine learning methods and summarized our review findings.

In supervised learning, a machine learning model is developed using both input data and target data (i.e. labelled outcome). The most common uses of supervised learning are prediction and classification. For example, a model that predicted suicide intent was based on a set of utterances labelled by a team of expert annotators as mentioning suicide or not (Fitzpatrick et al., 2017). In contrast, unsupervised learning methods are conducted based solely on input data. Unsupervised models provide insights by grouping unlabelled data and extracting its underlying features. Common tasks for unsupervised learning are cluster analysis and dimensionality reduction. An example application is a topic extraction from a counselling dialogue (Imel et al., 2015).

Thirteen articles of twenty‐three used supervised machine learning algorithms. Among them, seven articles used neural networks and six articles used logistic regression. Nine articles reported using unsupervised machine learning methods. Among the unsupervised methods, six articles adopted latent Dirichlet allocation (LDA) and three articles applied lexicon‐based methods. Two articles implemented both supervised and unsupervised approaches. Imel et al. (2015) used both labelled and unsupervised LDA models to identify themes in a collection of counselling transcripts. The results obtained from an unsupervised model can be further used as input for a supervised task. Wiegersma et al. (2020) extracted features using the National Research Council, NRC (Mohammad & Turney, 2013), lexicon and applied these features to the hotspot classification task.

Five articles used a combination of machine learning and inferential statistics. More specifically, four articles conducted hierarchical linear models with the input data obtained from machine learning methods (Atzil‐Slonim et al., 2021; Halfon et al., 2021; Shapira et al., 2021; Syzdek, 2020). Hierarchical linear models were the choice of these studies because they could handle the data dependence problem arising from the nested structure of conversational data (i.e. sessions are nested within patients). One article (Fontao & Mergenthaler, 2008) used ANOVA to test the relationship between therapeutic factors and linguistic features of a dialogue.

##### Model validation

Cross‐validation refers to the process of testing the performance of a trained model against the new data (Chowdhury, 2005). To do so, a model is first trained on a part of the data (train set) and then tested against the other part (test set) to obtain measures of model performance. The single train–test split is the simplest cross‐validation method where the training–testing process is done only once. The K‐fold cross‐validation repeatably splits the data K times, updates the trained model using different parts of the data and tests their performance (Koul et al., 2018; Refaeilzadeh et al., 2009). The K‐fold method is more robust than a single‐split method (Chowdhury, 2005). Twelve of twenty‐three articles used cross‐validation to evaluate model performance. Ten articles used single‐split and two used K‐fold cross‐validation methods.

##### Performance measures

Fourteen articles reported performance metrics that are common in machine learning applications, including F1 score, accuracy, precision and area under the curve (AUC). These methods are based on a cross‐tabulation table (a.k.a., confusion table) with counts of true positives, true negatives, false positives and false negatives. Besides, seven articles reported the statistical significance of their results, with two being accompanied by an effect size measure.

### RQ3: Evaluation of study generalizability

Nine of forty‐one (22%) reviewed articles addressed the issue related to the generalizability of their results. Four articles evaluated the issue from the perspective of model validation. Seven discussed the threat to generalizability due to the limitations of their samples. Four articles mentioned how their research designs limited the generalizability. Two articles indicated that their results had good generalizability because of their quality data and study design.

Among the four articles that attempted to enhance generalizability via cross‐validation, Atkins et al. (2012) reported that ‘using the leave‐one‐out type of cross‐validation provides strong evidence for the generalizability of the model’ (p. 10). The other three articles directly regarded cross‐validation results as evidence (Flemotomos et al., 2021; Tavabi et al., 2020) or counterevidence (Wiegersma et al., 2020) for generalizability.

The small sample size or non‐representative sample is a known threat to generalizability, and seven papers acknowledged this limitation. The data used by Nitti et al. (2010) consisted of 40 counselling sessions from a single subject. Relatively small sample sizes were reported by Hoogendoorn et al. (2017) with data obtained from 69 patients and Goldberg et al. (2020) with 386 participants. Syzdek (2020) and Burkhardt et al. (2021) also raised the question of the unrepresented sample. For instance, Burkhardt et al. (2021) described the sample used in a study as ‘predominantly young and, considering that they used a paid online therapy service, presumably financially stable; in addition, 25% were residents of California or New York, and 77.81% were female’ (p. 12). They acknowledged that non‐represented demographic and geographic groups may differ in their language use and word choice, and, as a result, the lexicon constructed in their study may not be generalizable to a general population. One article reported the lack of validation on an external sample as a limitation of generalizability (Bantilan et al., 2020).

Four articles cautioned about the limitations of their research designs. Boldrini et al. (2018) pointed out that most psychoanalytic treatments used in their study were limited to a specific historical period and may not generalize well to the current treatment methods. In addition, Syzdek (2020) noted that the nature of sentiment can differ across types of psychotherapy. A similar concern was raised by Tay (2020), who analysed the relationship between linguistic patterns and therapeutic modality for only client‐centred therapy. Xu, Xu, et al. (2021) used Cantonese data and indicated that their model required further testing before it could be used for other languages and cultural settings.

Another issue is the use of data from sessions with trainee therapists instead of licensed therapists. Since large‐scale psychotherapy data are difficult to access due to privacy concerns and data collection costs, some researchers attempt to use any available counselling data. For example, Atzil‐Slonim et al. (2021) attempted to use data from psychotherapy sessions conducted as a part of a counsellor training programme. However, as the authors indicated, this action may have compromised the generalizability of their results.

Researchers in two articles stated that their results could be generalized to other contexts. Cariola (2015) indicated that the findings from their study of counselling transcripts could be generalized to other types of data, including written autobiographical memories. Hoogendoorn et al. (2017) believed a good generalization from German to English despite the differences between the two languages. However, these two studies did not provide extra evidence for their claims about generalizability.

### RQ4: Assessment of bias

Ten of forty‐one articles (24%) indicated the possibility of NLP biases. Among them, six articles addressed the issue of biases arising from annotations (label bias), three articles drew a connection between bias and model validation and two articles mentioned biases occurring during the model implementation.

Hovy and Prabhumoye (2021) pointed out that label bias might arise due to annotators' subjectivity and predispositions. Two of the six articles that addressed bias discussed label bias caused by annotators. For example, Flemotomos et al. (2021) noted that when metadata, that is, information about therapists being rated, were available to coders, it might have affected their labelling decisions. Authors suggested that when rating therapist abilities some of the coders might have scored therapists from well‐known health care centres higher due to their reputation. In Xu, Xu, et al.'s (2021) paper, they discussed the possibility of bias introduced by individual annotators and Atkins et al. (2014) suggested that individual annotator bias might explain why the model performance was poorer for the utterance‐level classification. These two articles attempted to minimize label bias by calibrating on unlabelled test data before coding (Flemotomos et al., 2021) and by using only licensed experts (Xu, Xu, et al., 2021).

Three articles addressed measurement bias arising from an unvalidated psychological scale, a diagnostic instrument (Hull et al., 2021) and a self‐report instrument (Goldberg et al., 2020; Shapira et al., 2021). Additionally, another three articles explored bias reduction via model validation. Halfon et al. (2021) alluded that cross‐validation allowed them to avoid subjective bias. Wiegersma et al. (2020) and Goldberg et al. (2020) reported that they used cross‐validation as a bias reduction method.

The implementation of the model, encompassing the utilization of the model and interpretation of results, may introduce another potential source of bias. To mitigate implementation bias, users were advised to apply NLP models in the same context (e.g. purpose and population) for which they were originally developed (Flemotomos et al., 2021; Tavabi et al., 2020). For example, Flemotomos et al. (2021) noted:

An automated system for psychotherapy quality assessment also needs to be adapted to the actual use case and it is essential that the final user be aware of the training conditions and the potential limitations which are due to condition mismatch (Flemotomos et al., 2021, p. 15).

## DISCUSSION

This study systematically examined the applications of NLP in counselling and psychotherapy over the past three decades. From a review of 41 articles, we identified the primary purposes, advancements, limitations and challenges of NLP applications. Additionally, we examined how issues of generalizability and bias had been addressed. Our findings revealed an increasing trend in the adoption of advanced machine learning methods, especially after 2020. The predominant purpose for utilizing NLP was automated labelling. Additionally, less than one‐third of the articles discussed the generalizability of the findings and/or the presence of bias in their NLP applications.

Among the four purposes identified for applying NLP in counselling and psychotherapy, more articles focused on automated coding. The choice of the method for automated coding depended on the type of coding task. Studies on utterance‐level classification generally chose supervised methods that could model complex relationships. In contrast, for session‐level automated coding, researchers often preferred unsupervised methods like latent Dirichlet allocation.

Some challenges stand out when conducting automated coding on counselling and psychotherapy data. While some studies were able to obtain good accuracy with automated coding (Ramakrishna et al., 2018; Tanana et al., 2016), many studies did not have the same success due to the use of unstructured and interactive transcripts data. Some of the reported challenges in handling conversational data included small and unrepresented samples, a large number of labels in the coding system and multilabel classification (Laricheva et al., 2022). More research is needed to resolve these challenges.

We also found that advanced machine learning techniques have become more popular in NLP applications for counselling data (vs. automated text analysis tools). After 2020, neural networks have become the most popular NLP algorithm. In 2021, five of the ten articles that we reviewed implemented neural‐based models. This finding is consistent with other reviews on machine learning and NLP in mental health (Aafjes‐van Doorn et al., 2021; Shatte et al., 2019; Su et al., 2020).

Our results also revealed that less than a third of the reviewed studies addressed the issues of generalizability and bias. We noticed the following common pitfalls: lack of adequate description of the samples, poor agreement in human annotation and violations of statistical assumptions (inattention to data distribution) when analysing quantified data. These pitfalls may lead to biased results. When the sample data for training are not carefully examined before NLP, it may introduce selection bias (Bender & Friedman, 2018) and lead to poor performance for certain demographic groups who are not well represented in the sample. However, only 6 of 41 articles provided the demographics of their sample. To avoid selection bias, we recommend researchers inspect their sample data before using NLP. Reports like participants' age, gender, race, education, state of residence and previous therapy experience can help inspect where the training data are representative of the target population.

Label bias may arise due to various factors, such as unclear annotation guidelines, the annotator's subjective interpretation of the rubrics and the training background of the annotators. Although six papers did discuss the issues of label bias in their work, they provided little information about their annotation procedures and the annotators. In our review, eight studies did not report any measures of annotation quality and one study reported a low level of interrater agreement (Cohen's kappa <0.5).^3^ Moreover, some papers only used a single annotator (Atkins et al., 2014; Fontao & Mergenthaler, 2008; Tanana et al., 2021). Human judgement is highly susceptible to subjectiveness; bias is almost inevitable when labelling by a single annotator (Amidei et al., 2020). Therefore, a minimum of two raters is recommended.

Inappropriate use of statistical techniques, such as machine learning models, can also introduce biases in NLP. Three studies reported using data that violated the normality assumptions required by their chosen statistical methods (Cardazzone et al., 2021; Fontao & Mergenthaler, 2008; Hoogendoorn et al., 2017). Other data complexities, such as class imbalance (very skewed distribution between labels) can greatly affect analysis results if they are not addressed properly (Subramanian et al., 2021). All the above‐mentioned pitfalls can lead to biased or non‐generalizable findings and should be cautioned when using NLP for research or application in counselling and therapy.

## CONCLUSION

Our study offered an updated review of NLP practices for counselling and psychotherapy applications, which includes advancements, limitations and gaps in NLP applications. In particular, we drew special attention to the issues of generalizability and bias when applying NLP to highly intricate and intense counselling conversation data. Poor generalizability and biases in research findings can create the majority fallacy and bring in more misunderstanding about certain individuals. If these issues are not addressed, or at least acknowledged, they could, sometimes, do more harm than good to these clients. By being aware of various sources of bias and by following the good practices described above, researchers can avoid pitfalls and apply NLP methods in psychological research appropriately.

## AUTHOR CONTRIBUTIONS

**Maria Laricheva:** Conceptualization; investigation; writing – original draft; methodology; validation; visualization; writing – review and editing; software; formal analysis; data curation; resources. **Yan Liu:** Conceptualization; investigation; funding acquisition; methodology; writing – review and editing; project administration; supervision; resources; validation. **Edward Shi:** Investigation; writing – review and editing; formal analysis; methodology; validation. **Amery Wu:** Writing – review and editing; resources; conceptualization.

## CONFLICT OF INTEREST STATEMENT

No conflicts of interest.

## Data Availability

The data that supports the findings of this study are available in Appendix A.

## Footnotes

^1^
Feasibility study refers to the assessment of the practicality of a previously proposed NLP system.

^2^
Play therapy refers to the method of therapy that uses play to help children express their feelings and deal with emotional problems.

^3^
Interrater agreement is a measure of annotation quality. It assesses the extent to which annotators assign the same labels or ratings to the same data points. Cohen's Kappa is one such measure that takes chance into account while computing the agreement.

## APPENDIX A

The detailed results of 41 articles.

**Table:**

Author and date of paper	Study purpose	Data used	Word embedding methods	NLP methods used	Statistical methods used	Validation methods used	Main findings	Evaluation metrics
Althoff et al. (2016)	To predict better conversational outcomes using NLP	Text‐based counselling, 80,000 conversations	TF‐IDF	VADER sentiment	Hidden Markov model, logistic regression; multilevel models	Cross‐validation	Developed new computational methods for large‐scale data sets, uncovering actionable conversation strategies linked to improved outcomes	AUC = 50%–70%
Atkins et al. (2014)	To compare session codes generated by NLP model with human judgement	Therapy transcripts, 148 sessions	–	Labelled LDA	–	Cross‐validation, train/test split	Identified that the ability of topic models to predict behavioural codes was directly related to how strong is its semantic content	Average AUC of 72%; ICC = 0.1–0.7
Atkins et al. (2012)	To investigate use of NLP for data exploration and summarization in psychotherapy	Therapy transcripts, 1486 sessions	–	LDA	Logistic regression	Cross‐validation	Generated codes had higher reliability with human codes for session tallies and also varied strongly by individual code	Accuracy from 65% to 75%
Atzil‐Slonim et al. (2021)	Extracting topics from psychotherapy sessions; classifying topics that predict alliance ruptures	Therapy transcripts, 873 sessions	–	LDA	Logistic regression; multilevel growth models	Cross‐validation	The results showed that topic models have strong concordance with some behavioural codes	65%–75% accuracy
Bantilan et al. (2020)	Detecting suicide content from written communication with a therapist	Text‐based counselling, 1864 conversations	TF‐IDF	Random Forest, XGBoost, MLP neural network	Logistic regression	Train/test split	The final NLP model identified risk‐related content from non‐risk content with good accuracy	ROC AUC = 73%–83%
Boldrini et al. (2018)	To explore the relationship between RF ratings and psychotherapy outcome	Therapy transcripts, 540 sessions	–	Computerized reflective functioning (CRF)	Generalized estimating equations (GEE)	–	RF is predictive of patient's functioning changes although it does not measure these changes.	*p* = .001
Burkhardt et al. (2021)	To investigate how linguistic indicators in online counselling sessions relate to depression symptomatology over time	Text‐based counselling, 10,000 conversations	–	LIWC	Multilevel models	–	LIWC markers of depression and novel linguistic indicators of activation were strongly associated with depression scores and longitudinal patient trajectories	*p* < .05; *R* ^2^ = .6–.8
Can et al. (2016)	To automatically tag MI codes, with a special focus on therapists' reflections	Therapy transcripts, 108 sessions	–	N‐grams, Hidden Markov, maximum entropy Markov	–	Cross‐validation	Presented a strong‐performing model for automatically detecting counsellor reflections in motivational interviewing that compared several sources of information drawn from session transcripts	F1 score = 81%, recall = 93%, specificity = 90%, precision = 73%
Cardazzone et al. (2021)	Evaluating the linguistic changes occurring during EMDR sessions in a patient suffering from anorexia nervosa	Therapy transcripts, 73 sessions	–	LIWC	ANOVA	–	The combined use of linguistic and statistical analyses demonstrated significant linguistic variations in some psychologically relevant LIWC categories used by the patient during the three phases of EMDR sessions	*p* < .001
Cariola (2015)	To study the correlation between use of words and body boundary finiteness in person‐centred therapy	Therapy transcripts, 240 sessions	–	LIWC	Spearman rank correlation	–	The results provided some confirmation that person‐centred psychotherapy would clarify patients' social value systems and behavioural expectations that are embodied in the increased body boundary finiteness	*p* < .01
Flemotomos et al. (2021)	To predict total CTRS score of a session (low/high) or predict each code in a multitask approach	Therapy transcripts, 1118 sessions +4268 transcripts for domain adaptation	BERT	BERT	–	Train/test split	Results suggest that a combination of different linguistic representations and machine learning techniques may be beneficial to take advantage of both localized and contextualized patterns	F1 = 72.6%
Fontao and Mergenthaler (2008)	Examining the relationships between therapeutic factors in group therapy and the language features of the group dialogue	Therapy transcripts, 42 sessions	–	Therapeutic cycle model	MANOVA, independent *t*‐test	–	The current findings show that the use of EAPs allows the identification of key moments in a group therapy process	*p* < .01
Gaut et al. (2017)	To automate coding process (session‐level and fine‐grained levels)	Therapy transcripts, 1181 sessions	TF‐IDF	Labelled LDA	Lasso regression	Cross‐validation	L‐LDA model has the potential to be an objective, scalable method for accurate automated coding of psychotherapy sessions that performs better than comparable discriminative methods at session‐level coding and can also predict fine‐grained codes	AUC = 66%–80%
Goldberg et al. (2020)	Predicting self‐reported therapeutic alliance	Therapy transcripts, 1235 sessions	TF‐IDF, sent2vec	–	Logistic regression + ridge regularization, correlation analysis	Cross‐validation	Results presented here suggest that ML models modestly predict alliance ratings, supporting the notion that ML may be a useful tool for examining alliances	MSE = 0.67, Spearman's *p* = .15
Halfon et al. (2021)	To classify affect expressions in child psychotherapy	Play therapy recordings, 148 sessions		Lexicon‐based methods, neural networks, Decision Tree, Random Forest	Linear and polynomial regression, SVR, multilevel models, correlation analysis	Cross‐validation, train/test split	Experimental results show significant associations between automated affect predictions and CPTI affect dimensions with small‐ to medium‐effect sizes	*p* < .01
Hoogendoorn et al. (2017)	Predicting treatment outcome based on email conversations	Text‐based counselling, 69 conversations	–	LDA	Logistic regression	Cross‐validation	While it is difficult to draw sound conclusions on specific predictors, some interesting correlations could be observed in all different categories of attributes, which were explainable from a therapeutic perspective	AUC = 83%, precision = 78%
Hull et al. (2021)	Identifying major themes and symptom clusters in the SMS text messages that patients send to therapists	Text‐based counselling, 219,000 conversations	TF‐IDF	–	Correlation analysis	–	Results show a significant increase in the incidence of COVID‐19‐related intake anxiety symptoms, but no significant differences in the incidence of intake depression symptoms	*p* < .001
Imel et al. (2015)	To investigate the use of topic models to distinguish between therapy approaches	Therapy transcripts, 1500 sessions	–	LDA, labelled LDA	–	Cross‐validation	Results showed that topic models identified clinically relevant content, including affective, relational and intervention‐related topic	Error of discrimination = 13%
Kahn et al. (2008)	Examine the relationship between a client's disclosure of emotional material in an analogue psychotherapy session and the depth and smoothness of that session	Therapy transcripts, 33 sessions	–	LIWC	Multilevel models	–	While controlling for client functioning, sessions in which clients disclosed more and used more positive‐emotion words in their disclosures were rated as having more depth	*p* < .001, *R*‐squared of .2–.3
Lepper and Mergenthaler (2005)	To identify and analyse critical moments in psychotherapy interaction that can be seen as contributing to the experience of cohesion	Therapy transcripts, single session	–	Therapeutic cycles model	*T*‐test	–	Study demonstrated that the features of the dynamics of the therapeutic talk can be mapped onto the cycles identified by the TCM	*p* < .001; Cohen's *d* from 0.73 to 5.74
Lepper and Mergenthaler (2007)	Locating clinically significant events in therapy	Therapy transcripts, 8 sessions	–	Therapeutic cycles model	Kruskal–Wallis test	–	Findings suggest that topic coherence is correlated with periods of high therapeutic productivity identified by the TCM	*p* < .001; Cohen's *d* from 2.13 to 2.57
McCarthy et al. (2011)	To investigate therapist–patient dynamic processes across 16 sessions of psychotherapy	Therapy transcripts, 16 sessions	–	Therapeutic cycles model	Mann–Whitney *U* tests, correlation analysis	–	The TCM identified interventions of the therapist instigating change in emotion–abstraction patterns	*p* < .05; Cohen's *d* from 1.52 to 2.19
McCarthy et al. (2014)	To examine how therapist–patient emotional and cognitive dialogue influences therapeutic change for the patient	Therapy transcripts, 20 sessions	–	Therapeutic Cycles Model	Mann–Whitney *U* tests, Friedman two‐way ANOVA, Conover non‐parametric method of multiple comparisons	–	Results showed that connecting cycle sequences of problem‐solving in the third hour predicted 12‐month clinical outcomes	*p* < .05; Cohen's *d* from 1.03 to 1.98
McCarthy et al. (2017)	To recognize significant change moments from transcripts	Therapy transcripts, 20 sessions	–	LIWC + therapeutic cycles model	–	–	Significant events were saturated with both positive and negative emotion words, particularly anger and sadness, and more cognitive insight words	*p* < .001
Nitti et al. (2010)	Proposing a method to distinguish between stages of therapy using transcripts	Therapy transcripts, 43 sessions	–		Sequence analysis, cluster analysis, neural networks	Train/test split	The DFA can contribute to elaborating and validating a general model of the psychotherapy process (TSSM)	*R*‐squared of .1; *p* < .05
Pfäfflin et al. (2005)	To examine change processes within individual therapy sessions and during the course of treatment	Therapy transcripts, 110 sessions	–	Therapeutic cycles model	Mann–Whitney *U* test	–	The predicted relationship between the phases of the TCM and scores on the respective scales of the clinical ratings were supported	*p* < .001
Qiu and Tay (2021)	To explore how language use in psychotherapy is associated with different therapeutic approaches and therapeutic roles	Therapy transcripts, 155 sessions	–	LIWC	Multilevel models	–	The findings underline professional knowledge and institutionalized roles as key factors influencing the use of therapeutic language	*p* < .001, *R*‐squared of .2–.5
Ramakrishna et al. (2018)	To present a model for humour recognition in motivational interviewing‐based psychotherapy sessions	Therapy transcripts, 353 sessions	LSTM, GloVe	LSTM, support vector machine	–	Cross‐validation	The end‐to‐end model showed higher performance than the glove‐based model even in the absence of any context by making use of task‐specific embeddings	Accuracy = 90%, F1 = 70%, precision = 90%, recall = 60%
Rentscher et al. (2017)	To examine first‐person plural and singular pronouns as linguistic markers of communal coping and behavioural predictors of treatment outcome	Therapy transcripts, 584 sessions	–	LIWC	Logistic regression	–	Results strengthen evidence for the prognostic significance of spouse behaviour for patient health outcomes and for communal coping (indexed via pronoun use) as a potential mechanism of change in couple‐focused interventions for health problems	*p* < .001
Shapira et al. (2021)	To examine whether linguistic features were associated with clients' experiences of distress and outcome	Therapy transcripts, 729 sessions	–	Lexicon‐based methods	Multilevel models	–	The findings provide preliminary support for the association between clients' linguistic features and their fluctuating experience of distress	*p* < .05, Cohen's *d* of 0.01
Sonnenschein et al. (2018)	Analysing the verbal behaviour of patients with mood or/and anxiety disorders during psychotherapy	Therapy transcripts, 85 patients	–	LIWC	MANOVA	–	Differences between the three diagnostic groups were found in verbal utterances related to sadness. No differences were found for first‐person‐singular pronouns and content‐free fillers	*p* < .05
Soriano et al. (2017)	To identify semantic themes that differentiated couples with successful and unsuccessful treatment outcomes	Therapy transcripts, 584 sessions	–	W‐matrix	Chi‐square test	–	Results emphasize the role of spouse behaviour indexed via language use in alcohol treatment outcomes	*p* < .01
Syzdek (2020)	To evaluate change in sentiment within and across therapy sessions and the relationship between therapist and client sentiment	Therapy transcripts, 20 courses with at least 5 sessions each	–	Sentiment analysis	Multilevel models	–	Results indicate that there was significant interaction effect, with increases in positive sentiment across therapy sessions, while positive sentiment tended to decrease within sessions	*p* < .001
Tanana et al. (2016)	Automating coding for motivational interviews	Therapy transcripts, 341 sessions	GloVe	Discrete sentence features model, recurrent neural networks	–	Cross‐validation, train/test split	Results show that the DSF model generally had slightly better performance compared to the RNN model	Cohen's kappa >0.6
Tanana et al. (2021)	To develop a model for identification of emotions	Therapy transcripts, 97,497 utterances	BERT	LIWC; BERT, recurrent neural networks; maximum entropy model	–	Train/test split	Our findings revealed that the unigram sentiment model outperformed LIWC, and ultimately BERT outperformed both models	Cohen's kappa = 0.3–0.48
Tavabi et al. (2020)	Analysing behavioural cues language during a therapy session	Therapy transcripts, 219 sessions	BERT	LIWC, neural networks	Hierarchical ANOVA	Train/test split	The analysis demonstrates that MI codes may be estimated using clients' textual utterances along with preceding textual context from both the therapist and client	F1 = 72%
Tay (2020)	To explore ‘language styles’ in psychotherapy across sessions in time	Therapy transcripts, 80 sessions	–	LIWC	Cluster analysis	–	The study demonstrated the combination of computerized text, clustering and manual analysis to gain insights into evolving language styles in psychotherapy	*p* < .05
Tonti and Gelo (2016)	To investigate the relationship between a client's rate of speech (ROS) and emotional–cognitive regulation during a psychotherapy session	Therapy transcripts, single session	–	Therapeutic cycles model	ANOVA; Shapiro–Wilk test; correlation analysis	–	The results support the hypothesis that a significant reduction in the client's ROS may be a reliable marker of in‐session change processes	*p* < .001
Wiegersma et al. (2020)	To develop a model to automatically recognize traumatic memories based on text and speech features	Therapy transcripts, 20 patients	TF‐IDF	LIWC; support vector machine; lexicon‐based methods	–	Train/test split	Results show that the selected text and speech features could clearly distinguish between hotspots and non‐hotspots in the current data set but will probably not recognize hotspots from new input data very well	Precision = 60%; recall = 100%; F1 = 75%; accuracy = 75%
Xiao et al. (2016)	To propose a speech and language technology‐based system to automate the assessment of therapist empathy	Therapy transcripts, 1200 sessions	–	Maximum‐likelihood regression, maximum entropy model	–	–	The results show that the system provides useful information that can contribute to automatic quality insurance and therapist training	Accuracy = 71%–89%, RMSE = 1.27–1.73
Xu, Xu, et al. (2021) and Xu, Liu, et al. (2021)	Detecting suicide risk in text‐based counselling services	Text‐based counselling, 5682 online conversations	word2vec; TransD	BiLSTM	–	Train/test split	The proposed model significantly outperformed standard NLP models, demonstrating good translational value and clinical relevance	Precision = 87%, c‐statistic = 82%

Abbreviations: ANOVA, analysis of variance; AUC, area under ROC curve; BERT, bidirectional encoder representations from transformers; BiLSTM, bidirectional long short‐term memory neural network; CTRS, Cognitive Therapy Rating Scale; ELM, extreme learning machine; EMDR, eye movement desensitization and reprocessing; ICC, intraclass correlation; LDA, latent Dirichlet allocation; LIWC, linguistic inquiry and word count; LSTM, long short‐term memory; MANOVA, multivariate analysis of variance; MI, motivational interviewing; MLP, multilayer perceptron; NLP, The natural language processing; RMSE, root‐mean‐square error; ROC, receiver operating curve; SVR, support vector regression; TF‐IDF, term frequency–inverse document frequency; XGBoost, extreme gradient boosting.
